# Training Reveals the Sources of Stroop and Flanker Interference Effects

**DOI:** 10.1371/journal.pone.0076580

**Published:** 2013-10-11

**Authors:** Antao Chen, Dandan Tang, Xuefei Chen

**Affiliations:** 1 Key Laboratory of Cognition and Personality (Ministry of Education) and School of Psychology, Southwest University, Chongqing, China; 2 School of Psychology, Liaoning Normal University, Dalian, Liaoning, China; 3 Teacher Education School, Qujing Normal University, Qujing Yunnan, China; University G. d’Annunzio, Italy

## Abstract

In the field of cognitive control, dimensional overlap and pathway automaticity are generally believed to be critical for the generation of congruency effects. However, their specific roles in the generation of congruency effects are unclear. In two experiments, with the 4∶2 mapping design, we investigated this issue by examining the training-related effects on congruency effects (the Stroop interference effect and the Flanker interference effect in Experiments 1 and 2, respectively) normally expressed as incongruent minus congruent difference and on their subcomponents (the stimulus interference and response interference). Experiment 1 revealed that the stimulus interference in the Stroop task, wherein the task-relevant (printed color of word) and the task-irrelevant (semantics of word) dimensions of the stimuli were processed in different pathways, was present during early training but was virtually eliminated at the late stage of training. This indicates that the two dimensions overlap at the early stage but separate at the late stage. In contrast, Experiment 2 showed that the response interference in a variant of the Flanker task, wherein the task-relevant (central color word printed in black font) and the task-irrelevant (flanking color words printed in black font) dimensions of the stimuli were processed in the same pathway, was enhanced after training. This indicates that the enhanced automaticity of irrelevant-dimension processing induces stronger response competition, which therefore results in the larger response interference. Taken together, the present study demonstrates that (1) dimensional overlap is necessary for the generation of congruency effects, (2) pathway automaticity can affect the size of congruency effects, and (3) training enhances the degree of automatic processing in a given pathway.

## Introduction

Congruency effects (e.g., the Stroop, Simon, and Flanker effects) are widely used for investigating the cognitive control that is necessary to overcome the prepotent response tendencies in order to execute a less familiar response [1], [2], [3], [4]. According to the task requirements, the stimuli (or features) in the congruency tasks, i.e., the Stroop [5], Flanker [6], and Simon [7] tasks, can be divided into two dimensions: the task-relevant dimension and the task-irrelevant dimension. For example, in the Stroop task, stimuli are typically colored words with color meanings (e.g., RED is printed in blue font), where the task-relevant dimension is the printed color of word and the task-irrelevant dimension is the semantics of word. In the Simon task, stimuli typically consist of colored shapes (e.g., red and blue circles) that are presented either to the left or to the right of a central fixation, where the task-relevant dimension is the color of the circle and the task-irrelevant dimension is the position of it. In the Flanker task, stimuli typically consist of strings of letters (e.g., a central letter and four flanking letters, HHSHH), where the task-relevant dimension is the central letter and the task-irrelevant dimension is the flanking letters. In these tasks, participants are usually instructed to respond to the task-relevant dimension of stimulus while ignoring the task-irrelevant dimension of it, e.g., to press a left button when the middle letter is S and to press a right button when the middle letter is H in the Flanker task. According to the compatibilities of the task-relevant and task-irrelevant dimensions, the trial types consist of the congruent and incongruent trials. The task-relevant and task-irrelevant dimensions are compatible in the congruent trial types, whereas they are incompatible in the incongruent trial types. The body of empirical studies shows that performance [reaction times (RTs) and/or error rates] is generally worse in the incongruent relative to congruent trial types. Typically, the congruency effects can be quantified by the performance difference between the incongruent and the congruent trial types.

One basic issue about cognitive control is how to understand the generation of the congruency effects. In the relevant literature, the influential theoretical models used for understanding this issue are the parallel distributed processing (PDP) model [8], [9], [10] and the dimensional overlap (DO) model [11]. The PDP model [9] defines two processing pathways (the task-relevant and task-irrelevant pathways). They are used for processing the task-relevant and task-irrelevant information, respectively; and both converge on a common response mechanism. Each processing pathway comprises a set of input units, intermediate units, and output units. Specifically, each input unit in a given processing pathway is projected to the intermediate units in that pathway. Simultaneously, the intermediate units from both processing pathways are projected to all of the output units, where a task-relevant response may be produced. That is, the task-relevant and task-irrelevant information are processed by activating separate input and intermediate units in the given processing pathway. Activation then gradually propagates to the output units. The task-relevant response is produced if activation has been sufficiently accumulated from the output units of the task-relevant pathway to exceed a response threshold. Accordingly, the congruency effects stem from (1) the competition of the task-relevant and task-irrelevant responses in the output units and (2) processing advantage (higher automaticity) of a given pathway, usually considered to be the task-irrelevant pathway.

As regards the DO model [11], it is well known that it is initially proposed as a taxonomy of the congruency effects to account for various stimulus-response compatibility effects, considering the stimulus-stimulus (S–S) and stimulus-response (S–R) interference effects. It is based on similarity, which occurs in perceptual, structural, and conceptual attributes between the stimuli sets, and/or between the stimulus and response sets. Any overlap between the task-relevant and the task-irrelevant stimulus sets, between the task-relevant stimulus and the response sets, or between the task-irrelevant stimulus and the response sets automatically activates its more strongly associated response in the set. Therefore, the congruency effects stem from the interplay (1) between the task-relevant and the task-irrelevant stimulus processing and (2) between the automatically activated response and a controlled response tendency (reflecting the S-R mapping instructions).

On one hand, although the two models use different terms, they are in agreement that the overlap (intersection) as well as interplay between the task-relevant and the task-irrelevant processing are important for the generation of the congruency effects. On the other hand, the PDP model emphasizes that (1) the task-irrelevant pathway compared to the task-relevant pathway has a processing advantage and (2) the congruency effects stem from the response competition between the task-relevant and the task-irrelevant pathways in the output units; by contrast, the DO model emphasizes that (1) overlap in any given S-S sets and/or S-R sets may trigger the congruency effects and (2) the processing efficiency is comparable between the task-irrelevant and the task-relevant dimensions.

It has been known that both the Stroop and the Flanker interference effects consist of two kinds of interference [12], [13]: the stimulus interference and the response interference [6], [14], which are believed to occur in the stimulus process and the response output stages, respectively. A lot of studies (e.g., [14], [15], [16], [17], [18], [19]) have adopted the 4∶2 mapping design to separate the two types of interference. In the design, there are four kinds of task-relevant stimuli and two kinds of responses. Specifically, two of the task-relevant stimuli are mapped onto one response key and the other two are mapped onto another response key [6]. For example, in the Stroop task, participants may be told to press one key if the printed color of word is blue or green, and to press another key if the printed color of word is red or yellow [14], [15], [18], [20], [21]. This manipulation allows for three trial types: the congruent (CO; the semantics and the printed color of word are congruent, e.g., ‘RED’ is printed in red font), the stimulus incongruent (SI; the semantics and the printed color of word are incongruent, but the activated responses between the two pathways are compatible, e.g., ‘YELLOW’ is printed in red font); and the response incongruent (RI; the semantics and the printed color of word are incongruent, and the induced responses between the two pathways are incompatible, e.g., ‘BLUE’ is printed in red font). The RI trial type is assumed to involve both kinds of interference, the SI trial type is suggested to contain only the stimulus interference, and the CO trial type contains neither. Therefore, the Stroop interference effect can be separately calculated by the RT differences between the SI and the CO trial types to quantify the stimulus interference, and between the RI and the SI trial types to quantify the response interference. The same design can be extended to the Flanker task to divide the Flanker interference effect into the stimulus and response interference [6], [17]. However, the Simon interference effect cannot be differentiated with this design, because the limitation of S-R set (two stimuli and two responses) [22].

As proposed by the PDP model, automatic processes are continuous and training can change the automaticity of a processing pathway, training is a useful method to decompose the sources of the Stroop interference effect and to reveal the dynamic changes in each of the interference [16]. For example, in the Stroop task, although the word reading is automatic and dominant relative to the color naming, training can change the automaticity of the color naming pathway and therefore influence the interference effect [23], [24]. Indeed, some studies find that training may enhance the processing automaticity of the task-relevant dimension while suppress that of the task-irrelevant dimension [25], [26], [27]. However, the training-related effects on the Flanker interference effect are not clear.

On the basis of the DO taxonomy, in the Flanker task, since both the task-relevant and the task-irrelevant stimuli are processed in the same pathway (e.g., ‘HHSHH’, the letters ‘H’ and ‘S’ belong to the same category), the two dimensions always overlap and the task-irrelevant dimension has no processing advantage over the task-relevant dimension. In this case, the Flanker interference effect is probably due to the dimensional overlap between the task-relevant and the task-irrelevant stimuli rather than the processing advantage of the task-irrelevant dimension. However, in the Stroop task, since stimulus comprises the printed color and semantics of word, they are processed in the different processing pathway and the word reading has a processing advantage over the color naming [28], [29]. In this case, the Stroop interference effect can seemingly be explained by the PDP model. That view is confirmed by recent experimental evidence, suggesting that the two dimensions are processed by separate neural structures and pathways [16], [26], [30], [31]. Hence, we speculate on that the nature of the Stroop and the Flanker interference effects is different, and the training-related effects on them may be distinct.

The present study uses the 4∶2 mapping design, where participants will perform a considerable number of trials, which allows us to examine the training-related effects on the congruency effects and on their two subcomponents (both the stimulus interference and the response interference). In the Stroop task (Experiment 1), the task-relevant and task-irrelevant dimensions are different in categories (color vs. word). According to the PDP model [8], which proposes that the word reading relative to the color naming is more automatic, and the automaticity can be viewed as a continuous phenomenon that varies with training, we predict a reduced Stroop interference effect that will be embodied in the reduction (even elimination) of stimulus interference and response interference with training because of the enhanced automaticity of color naming pathway and the relatively suppressed automaticity of word reading pathway [26], [27]. By contrast, in a variant of the Flanker task (Experiment 2), as both the task-relevant and the task-irrelevant dimensions of stimulus belong to the same category (center word vs. flanking words) and the word reading is very automatic, there is no processing advantage between them. Based on the DO model [11], as the dimension of flanking words always overlaps with that of center word, the processing of flanking words automatically activates the corresponding response that interferes with the correct response made according to the center word in the RI trials but facilitates those in both the SI and the CO trials. Here, we infer that with training, (1) the stimulus interference may keep stable because the training-related effects on the processing of center word and flanking words always are the same but (2) the response interference may be increasing due to the relatively impaired responses in the RI trials and the facilitated responses in the SI trials. These combined effects will result in an increasing Flanker interference effect with training.

## Experiment 1

Experiment 1 adopted the 4∶2 mapping design and the Stroop task to investigate the training-related effects on the Stroop interference effect and on the subcomponents (both the stimulus interference and the response interference). Participants firstly performed an eight-trial practice block before completing five training blocks (192 trials each). The initial minimal practice ensured that all participants had limited experience of performing the Stroop task and acclimated to the task conditions before the training experiment. Under this condition, their performance during the experimental phase could adequately reflect the training-related effects. In addition, the trail proportion of CO:SI:RI was 2∶1:1, which was in line with the previous studies [17], [18] and allowed the results of the present study to be compared with theirs.

### Method

#### Participants

Twenty-one university students (16 females, mean age = 21 years) participated in this experiment for monetary compensation. All participants had normal or corrected-to-normal vision, and had not performed the Stroop task previously. Approval of the study was made by the Human Research Ethics Committee of the Southwest University of China, and all participants provided written informed consent.

#### Apparatus and stimuli

The stimuli were presented using E-Prime software (Psychology Software Tools, Inc. Pittsburgh, PA) on a 17-in computer monitor. Responses were registered using a standard QWERTY keyboard. Participants sat approximately 50 cm away from the screen. Four words (RED, YELLOW, BLUE, and GREEN) were used (in Chinese with Song Ti font). These words were printed in red, yellow, blue, or green font. The RGB values for the stimulus colors were 255, 0, 0 (red); 0, 255, 0 (green); 0, 0, 255 (blue); and 255, 255, 0 (yellow), respectively.

#### Procedure

Instructions were presented on the computer screen, which informed participants that a colored word would be presented in each trial and they required to identify the printed color while ignoring the semantics of word. Each trial started with a blank fixation for 300–500 ms (the interval varied randomly), which was presented on a gray background. Then, a colored word was presented and remained on the screen until a response was made. Lastly, a grey screen was presented for 300–500 ms (the interval varied randomly). Participants were instructed to press the “Q” key with the left index finger if the printed color of word was red or yellow and to press the “P” key with the right index finger if the printed color of word was green or blue. In addition, participants were instructed to perform the task as fast as possible without sacrificing accuracy. After performing an eight-trial practice block, participants completed five training blocks; each block included 192 trials (96 CO, 48 SI, and 48 RI trials; presented randomly). There was a 2-minute break between blocks.

### Results

The mean RTs and error rates in the CO, SI, and RI trials for each participant and each block were computed separately. For the analysis of the RTs, all incorrect trials (8% of all trials), and trials (1.61% of all trials) in which the RTs were shorter than 150 ms or longer than 1,500 ms (avoiding the aberrant data due to (1) participants did not pay attention to the stimulus, which might result in slower RTs than 1,500 ms and (2) participants made responses arbitrarily, which might results in faster RTs than 150 ms) were excluded as outliers. In total, this step resulted in removing 9.61% of all trials.

To analyze the overall Stroop interference effect, we conducted a one-way repeated-measures analysis of variance (ANOVA) for the RTs in the CO, SI, and RI trials. The results revealed a significant RT difference among the CO, SI, and RI trials, *F*(2,40) = 53.33, *p*<0.001, *η* ^2^ = 0.73. Post hoc test revealed significantly increasing RTs in the CO, SI, and RI trials, *p*s <0.01, which indicated a significant Stroop interference effect. The mean RT results for Experiment 1 were displayed in Table 1. As could be seen in Table 1, the overall Stroop interference effect was gradually reduced with training.

**Table 1:** The mean RTs (ms) as a function of block and trial type in Experiment 1.

Types	Block 1		Block 2		Block 3		Block 4		Block 5	
	*M*	*SD*	*M*	*SD*	*M*	*SD*	*M*	*SD*	*M*	*SD*
CO	523	54	485	42	480	41	472	34	458	31
SI	544	61	493	51	482	40	472	44	462	32
RI	578	85	515	53	503	46	496	55	480	43

*Note:* CO is congruent, SI is stimulus incongruent, and RI is response incongruent; M is mean, SD is standard deviation. *N* = 21.

Then, the RT and error rate data were analyzed using the two-way repeated-measures ANOVA with the following variables: trial type (CO, SI, and RI trials) and block (5 blocks). Greenhouse-Geisser corrections were applied for the adjustment of degrees of freedom because these within-participants factors consisted of more than two levels. The results revealed significant main effects of trial type, *F*(2,40) = 53.33, *MSe* = 27,777, *ε* = 0.73, *p*<0.001, and block, *F*(4,80) = 38.60, *MSe* = 61,935, *ε* = 0.49, *p*<0.001. More importantly, the interaction between trial type and block was also significant, *F*(8,160) = 3.51, *MSe* = 1,101, *ε* = 0.47, *p*<0.01. The significant interaction indicated that training resulted in different effects on the trial type.

**Figure 1:**
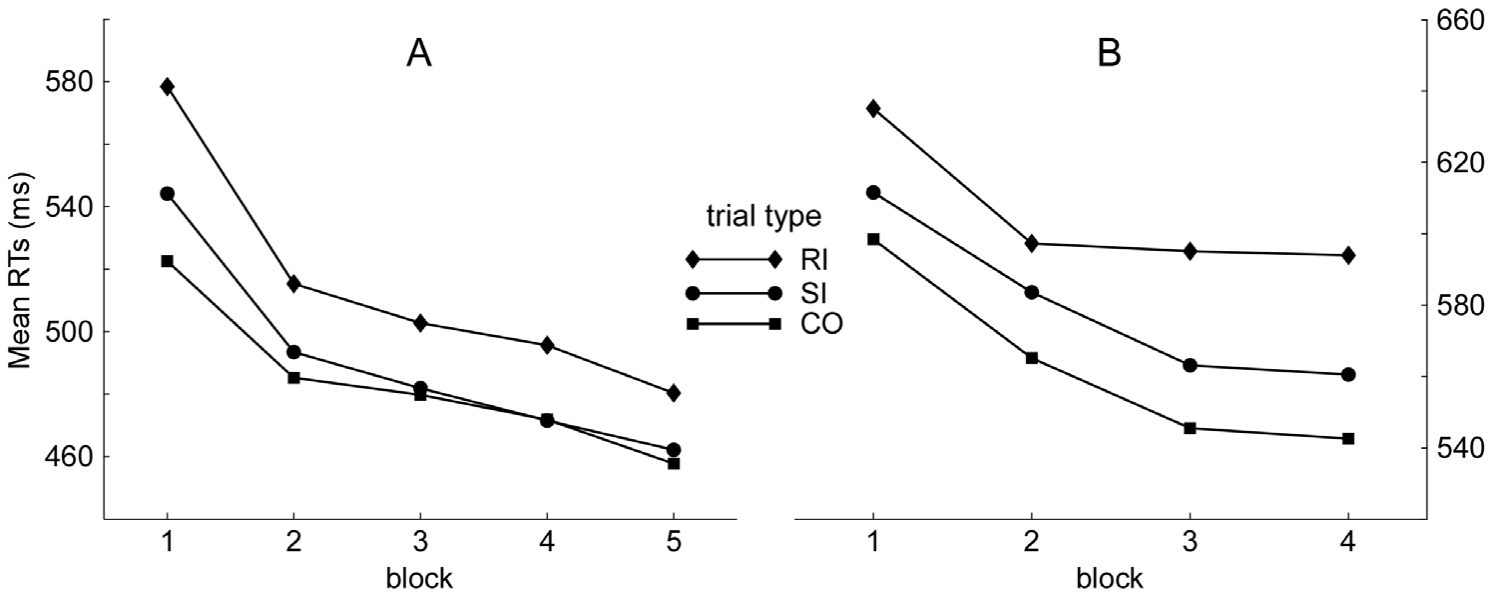
The mean RTs as a function of block and trial type in Experiments 1 and 2. Panel **A** (Experiment 1) indicates that the stimulus interference (RT_SI-CO_) is sharply reduced and almost eliminated with training in the Stroop task. In contrast, the response interference (RT_RI-SI_) starts to decrease in the second block, and is stable with the following training. Interestingly, Panel **B** (Experiment 2) indicates that (1) the stimulus interference starts increasing in the second block, and is stably present across the following training and (2) the response interference is decreased in the second block, but increases in the following training in the Flanker task. *Note:* CO, SI, and RI are congruent, stimulus incongruent, and response incongruent, respectively.

A main aim of the present study was to examine how the Stroop interference effect (i.e., stimulus and response interference) varied with training. Fig. 1A showed the training-related effects on the CO, SI, and RI trials. A two-way repeated-measures ANOVA was carried out with block (5 blocks) and trial type (including only the CO and SI trials) as variables to reveal the training-related variation of the stimulus interference. The results revealed significant main effects of both block and trial type, *F*(4,80) = 33, *MSe* = 90,268, *ε* = 0.45, *p*<0.001, and *F*(1,40) = 87.50, *MSe* = 50,720, *ε* = 1, *p*<0.001. Moreover, their interaction was also significant, *F*(4,80) = 6.11, *MSe* = 4,228, *ε* = 0.58, *p*<0.001. Those indicated that training-related effects on the SI and CO trials were different. A similar ANOVA was carried out with block (5 blocks) and trial type (including only the SI and RI trials) as variables to reveal the training-related variation of the response interference. The results demonstrated significant main effects of both block, *F*(4,80) = 35.45, *MSe* = 102,076, *ε* = 0.51, *p*<0.001, and trial type, *F*(1,20) = 39.5, *MSe* = 29,862, *ε* = 1, *p*<0.001. However, their interaction was not significant, *F* <1. Those indicated that training-related effects on the SI and RI trials were almost the same.

To clearly reveal the dynamic changes of the stimulus interference and response interference with training, the follow-up paired-samples *t* test was run in the five training blocks, respectively (Table 2). The results showed that the response interference (RI-SI) was significantly present in each block, despite there were sharp reduction in this effect in block 2 compared to block 1. However, the stimulus interference (SI-CO) was significantly present in block 1 but absent from block 2 to block 5.

**Table 2:** The results of test statistics (pair-wise *t* tests, two-tailed) for each block in Experiment 1.

Types	Block 1		Block 2		Block 3		Block 4		Block 5	
	D(SD)	*t*(20)	D(SD)	*t*(20)	D(SD)	*t*(20)	D(SD)	*t*(20)	D(SD)	*t*(20)
RI-SI	34(40.09)	3.91**	22(31.29)	3.20**	21(21.48)	4.44**	24(25.6)	4.32**	18(32.51)	2.56*
SI-CO	22(20.97)	4.73**	8(21.07)	1.80	2(21.91)	0.44	0(21.55)	0.06	4(12.01)	1.70

*Note.* *p<0.05, **p≤0.01, *N* = 21; D is the RT difference (ms) between the RI and the SI trials, or between the SI and the CO trials; SD is standard deviation; CO is congruent, SI is stimulus incongruent, and RI is response incongruent.

For the error rates, the two-way repeated-measures ANOVA, with the following variables: trial type (CO, SI, and RI trials) and block (5 blocks), revealed a significant main effect of trial type, *F*(2,40) = 28.50, *MSe* = 2.4, *ε* = 0.70, *p*<0.001, but neither significant main effect of block nor significant interaction were found, *F*s <1.9. Wherein, the error rates were 1.86%, 1.71%, and 4.43% for the CO, SI, and RI trials, respectively. The results revealed that training had little effect on error rates, and therefore the error rate data were not analyzed further.

### Discussion

Experiment 1 revealed a gradually reduced Stroop interference effect that was embodied in an elimination of the stimulus interference and a reduction (but still stable appearance) of the response interference with training (Tables 1 and 2). That was in line with the PDP model, and supported the prediction that the reduced Stroop interference effect would be embodied in the gradual reduction of both the stimulus interference and response interference with training.

The present results manifested that the training-related modulation on the stimulus and response interference was different. With training, the processing efficiency of color-naming pathway was significantly promoted but that of word-reading pathway was influenced little [25], [26], [27]. However, a potential factor, word reading and color naming were implemented at different cognitive levels, might confound the present results. To address the question, Experiment 2 adopted the same design in a variant of the Flanker task, in which both the task-relevant and the task-irrelevant stimulus dimensions (center word vs. flanking words) were processed at the semantic level. Meanwhile, we can also investigate the training-related effects on the Flanker interference effect and on the subcomponent (both the stimulus interference and the response interference).

## Experiment 2

The aims of Experiment 2 were to (1) eliminate the possible confounding that word reading and color naming were implemented at different cognitive levels in Experiment 1 and (2) investigate the dynamic changes of the stimulus interference and the response interference in the Flanker task. Experiment 2 was the same as Experiment 1, except that the Stroop task was replaced with a variant of the Flanker task, wherein both the task-relevant and the task-irrelevant dimensions (words) were processed at the semantic level. The stimuli consisted of horizontally arranged word triads, similar to those used in the study of Zhang and Kornblum [32]. For each trial, participants were asked to respond based on the identity of semantics of the middle word but to ignore that of the flanking words. The words (RED and YELLOW) were assigned to a left response, and the words (BLUE and GREEN) were assigned to a right response. Accordingly, there were three trial types. In the CO trial type, all three words were identical (e.g., BLUE-BLUE-BLUE). In the SI trial type, the flanking words differed from the middle word, but they were assigned to the same response (e.g., RED-YELLOW-RED). In the RI trial type, the flanking words differed from the middle word, and they were assigned to different responses (e.g., RED-BLUE-RED). Note, all words were printed in Chinese in black font, and there were no dashed lines between two neighboring words in the actual experiment.

### Method

#### Participants

Fifty-one university students (26 females, mean age = 22 years) participated in this experiment for monetary compensation. All participants had normal or corrected-to-normal vision and had never performed the Flanker task previously. Approval of the study was made by the Human Research Ethics Committee of the Southwest University of China, and all participants provided written informed consent.

#### Apparatus and stimuli

The apparatus were the same as those used in Experiment 1. Participants sat approximately 50 cm away from the screen. Four Chinese words (RED, YELLOW, BLUE, and GREEN) were used to form the word triads. These words were colored in black. In each word triad, the two flanking words were always identical; however, the middle word was either identical to the flanking words (e.g., BLUE-BLUE-BLUE) or different from them (e.g., RED-YELLOW-RED). Note that there were no dashed lines between two neighboring words in the actual experiment.

#### Procedure

The procedure was the same as Experiment 1 except the difference in experiment task. The participants were informed that a word triad would be presented in each trial and they were required to identify the semantics of the middle word while ignoring that of the two flanking words. They were instructed to press the “Q” key with the left index finger if the middle word was RED or YELLOW and to press the “P” key with the right index finger if the middle word was GREEN or BLUE. Participants were instructed to perform the task as fast as possible without sacrificing accuracy. An eight-trial practice block was implemented preceding four experimental blocks; each consisted of 192 trials (96 CO, 48 SI, and 48 RI trials; presented randomly). There was a 2-minute break between blocks.

### Results

The mean RTs and error rates in the CO, SI, and RI trials for each participant and each block were computed, respectively. For the analysis of the RTs, the same data elimination strategies were adopted as that adopted in Experiment 1. Therefore, there were 8.49% of all trials eliminated due to incorrect responses and 0.6% of all trials eliminated due to that the RT range was outside the time window of 150–1,500 ms. In total, 9.09% of all trials were eliminated as outliers.

To analyze the overall Flanker interference effect, we conducted the one-way repeated-measures ANOVA for the RTs in the CO, SI, and RI trials. The results revealed a significant RT difference among the CO, SI, and RI trials, *F*(2,100) = 39.1, *p*<0.001, *η* ^2^ = 0.44. Post hoc test revealed significantly increasing RTs for the CO, SI, and RI trials, *p*s <0.001, which indicated a significant Flanker interference effect. The mean RT results for Experiment 2 were displayed in Table 3, which showed an overall increasing Flanker interference effect with training.

**Table 3:** The mean RTs (ms) as a function of block and trial type in Experiment 2.

Types	Block 1		Block 2		Block 3		Block 4	
	*M*	*SD*	*M*	*SD*	*M*	*SD*	*M*	*SD*
CO	598	93	565	73	545	52	543	54
SI	612	97	584	85	563	69	561	60
RI	635	96	597	67	595	68	594	58

*Note:* CO is congruent, SI is stimulus incongruent, and RI is response incongruent; M is mean; SD is standard deviation. *N* = 51.

Then, the RT and error rate data were analyzed using the two-way repeated-measures ANOVA with the following variables: trial type (CO, SI, and RI trials) and block (4 blocks). Greenhouse-Geisser corrections were applied for the adjustment of degrees of freedom because these within-participants factors consisted of more than two levels. The results revealed the significant main effects of trial type, *F*(2,98) = 95.23, *MSe* = 91,122, *ε* = 0.97, *p*<0.001, and block, *F*(3,147) = 19.41, *MSe* = 77,615, *ε* = 0.69, *p*<0.001. More importantly, the interaction between trial type and block was also significant, *F*(6,294) = 3.66, *MSe* = 1,494, *ε* = 0.85, *p*<0.05. The significant interaction indicated that training resulted in different effects on the trial type (Fig. 1B).

To uncover the training-related effects on the Flanker interference effect, we carried out two follow-up two-way repeated-measures ANOVAs, respectively. The first ANOVA, with block (4 blocks) and trial type (including only CO and SI trials) as variables, revealed significant main effects of block and trial type, *F*(3,147) = 21.18, *MSe* = 85,703, *ε* = 0.71, *p*<0.001, and *F*(1,49) = 33.39, *MSe* = 28,140, *ε* = 1, *p*<0.001, respectively; however, their interaction was not significant, *F* <1. Those indicated that training almost resulted in the same effect on the SI and CO trial types. The second ANOVA, with block (4 blocks) and trial type (including only SI and RI trials) as variables, revealed significant main effects of block, *F*(3,147) = 13.93, *MSe* = 58,753, *ε* = 0.78, *p*<0.001, and trial type, *F*(1,49) = 58.32, *MSe = *65,583, *ε* = 1, *p*<0.001. And their interaction was also significant, *F*(3,147) = 3.97, *MSe* = 2,386, *ε* = 0.87, *p*<0.05. Those indicated that training-related effects on the SI and RI trials were different.

To clearly reveal the dynamic changes of the stimulus interference and the response interference, the follow-up paired-samples *t* test was performed in the four training blocks, respectively (Table 4). The results showed that both the stimulus interference (SI-CO) and the response interference (RI-SI) were significantly present across the four blocks. Then, we carried out the one-way repeated-measures ANOVA for the stimulus conflict (SI-CO) and response conflict (RI-SI) among the four blocks, respectively. For the comparison of stimulus conflict (SI-CO), the result showed that there was no significant difference among the four blocks, *F*(3,150) <1. For the comparison of response conflict (RI-SI), the difference was marginally significant among the four blocks, *F*(3,150) = 2.11, *p* = .07. Post hoc test revealed that the response conflict was marginally larger in block 4 than that in block 2, *p* = .08. The results indicated that the stimulus interference kept stable and the response interference increased with training (Fig. 1B).

**Table 4:** The results of test statistics (pair-wise *t* tests, two-tailed) for each block in Experiment 2.

Types	Block 1		Block 2		Block 3		Block 4	
	D(SD)	*t*(50)	D(SD)	*t*(50)	D(SD)	*t*(50)	D(SD)	*t*(50)
RI-SI	23(78)	2.96**	14(42.4)	2.36*	32(52.82)	5.12**	33(36.31)	5.60**
SI-CO	13(57.85)	2.97*	18(48.74)	3.21**	18(40.11)	3.31**	18(47.58)	3.37**

*Note.* *p<0.05, **p≤0.01, *N* = 51; D is the RT difference (ms) between the RI and the SI trials, or between the SI and the CO trials; SD is standard deviation; CO is congruent, SI is stimulus incongruent, and RI is response incongruent.

For the error rates, the two-way repeated-measures ANOVA, with the following variables: trial type (CO, SI, and RI trials) and block (4 blocks), revealed a significant main effect of trial type, *F*(2,98) = 36.07, *MSe* = 0.13, *ε* = 0.77, *p*<0.001, but there was neither significant main effect of block nor significant interaction between trial type and block, *F*s <1.45. Wherein, the error rates were 2.24%, 2.35%, and 3.90% for the CO, SI, and RI trials, respectively. The results were consistent with those of Experiment 1 (training had little effect on error rates), and therefore the error rate data were not analyzed further.

### Discussion

Experiment 2 revealed an increasing Flanker interference effect that was embodied in the constant stimulus interference and the increased response interference (despite slightly reduced in block 2 compared to block 1) with training (Tables 3 and 4). That was consistent with the DO model and our prediction that the increasing Flanker interference effect would be embodied in the increasing response interference after training; however, the stimulus interference would be little affected. Therefore, the results eliminated the confounding that word reading and color naming were implemented at different cognitive levels in Experiment 1. Meanwhile, the present results indicated that (1) training amplified the response interference but slightly affected the stimulus interference and (2) training-related effects on the Stroop and Flanker interference effects were distinctly different.

## General Discussion

In the present 4∶2 mapping design, the training-related effects on the Stroop interference effect, the Flanker interference effect, and their subcomponents (both the stimulus interference and the response interference) are investigated for the first time. The results show a decreasing Stroop interference effect (Experiment 1) but an increasing Flanker interference effect (Experiment 2) with training. In addition, there are some novel findings on the dynamic changes of the stimulus interference and the response interference with training due to the adoption of the 4∶2 mapping design in the classical Stroop task and variant of the Flanker task.

Firstly, in the Stroop task of Experiment 1, the stimulus interference is sharply reduced at the initial stage of training and virtually eliminated at last. The results manifest that with training, the automatics of color-naming pathway is enhanced and therefore the processing becomes more efficient for the color dimension [26], [27]. Specifically, the response interference is decreased but is still robust with training, indicating that the competition between the task-relevant and the task-irrelevant response may be intrinsic, which determines the persistent presence of the Stroop interference effect. Accordingly, with training, the reduction of the Stroop interference effect is due to the elimination of stimulus interference; however, after training, the persistence of the Stroop interference effect is attributed to the robust response interference (Tables 1 and 2). The results of Experiment 1 provide empirical support for the work of MacLeod, who considers that the reduction of the Stroop interference effect with training is due to the sharp decrease in the interaction between color and word processing, and the persistent competition in the response output pathways [23].

The experimental observation (the decreased Stroop interference effect) in Experiment 1 is in line with the hypothesis of PDP model [9]. According to the PDP model, the color naming and word reading have separate processing pathways. They are firstly processed by activating separate input and intermediate units. Activation then gradually propagates to the output units in both pathways. Because of the processing advantage of the word pathway over the color pathway, the response competition produces in this stage. Moreover, studies find that the strength of color processing pathway increases [9] and the color and word dimensions separate from each other [24] after adequate training. Thereby, training can strengthen the connection between the input and the intermediate units in the color-naming pathway. In this condition, the stimulus interference will decrease or even disappear with training. Accordant with this, the disappearance of stimulus interference in the present study is attributable to (1) the strengthened connection between the input and the intermediate units in the color-naming pathway and (2) the separation between the color-naming and the word-reading pathways. In addition, the persistence of the response interference after adequate training indicates that the competitions between the word and the color dimensions at the response output stage are still strong [27].

Secondly, in the variant of the Flanker task of Experiment 2, the overall Flanker interference effect increases with training; this is distinctly different from the finding in Experiment 1. Experiment 2 reveals that the stimulus interference keeps stable across training, which may indicate that the stimulus interference is intrinsic in the Flanker task; intriguingly, the response interference marginally increases after training, which indicates that the automatic response activation of flanking words is strengthened with training. Thus, the increased Flanker interference effect with training is attributed mainly to the increase of the response interference.

A critical feature of the present Flanker task is that the task-relevant and task-irrelevant dimensions of stimuli overlap within the same category (words). Thereby, the stimulus interference is determined by the semantic relations between the center and the flanking words rather than the competition strength between them [33], [34], [35], [36]. Under this condition, we observe the stable stimulus interference after training (Table 4), which eliminates the confounding factor of Experiment 1 (i.e., the difference in cognitive processing results in the abolished stimulus interference). Specifically, the present results can be explained by the DO model [11]. It proposes that similarity between the task-relevant and the task-irrelevant stimulus sets can result in dimension overlap in the stimulus processing stage, and the presentation of a stimulus automatically activates its task-irrelevant response that interferes with the task-relevant one. Accordingly, the increased response interference may be due to enhanced automaticity of the task-irrelevant S-R activation with training.

It is notable that the proportion of CO:SI:RI is 2∶1:1 for Experiment 1, which means that the probability of correct response will be 75% if participants make responses by adopting a word-reading strategy. Accordingly, one may argue that the abolishment of stimulus interference in Experiment 1 may be due to the specific proportion of the three trial types. If this is the case, the RTs in the SI trials will be almost the same as that in the CO trials throughout training. However, the results of Experiment 1 show dramatic reduced RTs for both the CO and the SI trials at the early stage of training, which is not consistent with the proportion interpretation. Moreover, the slightly enhanced stimulus interference in the Flanker task of Experiment 2 (adopting the same design with Experiment 1) also conflicts with the proportion interpretation. In addition, one previous study finds that a facilitation from the distractor enhances the early processing of the target stimulus [37]. However, if the participants tend to make a response based on word reading, larger response interference will be observed in Experiment 1 because word reading facilitates color naming in the SI trials but interferes with that in the RI trials. Conversely, the results of Experiment 1 show that the response interference reduces with training. Therefore, all results do not support the proportional interpretation.

Based on the findings of Experiments 1 and 2, the present study manifests that with training, (1) the Stroop interference effect reduces, which is embodied in the decrease or even elimination of stimulus interference and the stable presence of response interference; but (2) the Flanker interference effect increases, which is embodied in the stable presence of stimulus interference and the enhanced response interference. Accordingly, we propose that the nature of the Stroop and Flanker interference effects is different, and the training-related effects on them are distinctly different. Training enhances the processing efficiency of color-naming pathway and therefore slightly mitigates the response competitions between word-reading and color-naming pathways (Experiment 1). However, training affects little on the dimension overlap between the task-relevant and the task-irrelevant stimulus sets and enhances the associated response to the task-irrelevant stimulus dimension (Experiment 2). Thereby, on one hand, the present study provides an ideal method for revealing the sources of the Stroop and Flanker interference effects; on the other hand, the results further expand our understanding of the PDP and DO models.

In sum, the present study provides evidence that repeated training reduces the interference effects through bypassing the stimulus interference if the task-relevant and task-irrelevant dimensions do not share the process (consistent with the PDP). However, repetition has little effect in reducing the interference effects if there is an overlap in the stimulus processing (consistent with the DO model).
